# Cropping Up Crisis at the Nexus Between COVID-19 and Antimicrobial Resistance (AMR) in Africa: A Scoping Review and Synthesis of Early Evidence

**DOI:** 10.7759/cureus.21035

**Published:** 2022-01-08

**Authors:** Girma Gutema, Gadissa Homa

**Affiliations:** 1 Pharmacology, Rift Valley University, Adama, ETH; 2 School of Pharmacy, University of Oslo, Oslo, NOR; 3 Pharmacology, Hayat Medical College, Addis Ababa, ETH

**Keywords:** africa, health policy, antimicrobial resistance, impacts, covid-19

## Abstract

In this study, we aim to synthesize some evidence on the impacts that coronavirus disease 2019 (COVID-19) is having on the epidemiology of antimicrobial resistance (AMR) in Africa since it was declared a global pandemic by the WHO in March 2020.

A scoping review was undertaken by collecting and curating relevant resources from peer-reviewed articles and also from the gray literature. Mixed approaches of extracting data (qualitative and quantitative) were employed in synthesizing evidence, as suggested by the Health Evidence Network.

A model constructed based on the synthesis of early evidence available on the effects of factors linked to COVID-19 in impacting the evolution of AMR in Africa predicted that, in cumulative terms, those factors favoring the evolution of AMR outpace those disfavoring it by no less than three folds.

COVID-19 is likely fueling the evolution of AMR almost unhindered in Africa. Due to the recognition of this crisis, concerted efforts for resource mobilization and global cooperation are needed to tackle it.

## Introduction and background

The viral coronavirus disease 2019 (COVID-19) caused by severe acute respiratory syndrome coronavirus 2 (SARS-CoV-2), a novel variety of Sarbecovirus, was first detected in December 2019 in the central Chinese city of Wuhan [1]. From there, it rapidly spread globally, and by March 11, 2020, the World Health Organization (WHO) declared it a global pandemic [2,3]. Ever since, the pandemic is affecting all the micro and macro systems constituting the world order as we know it, in a way unprecedented in recent memory, so much so that its overall impacts are now thought to carry a tangible opportunity to bring about “the great reset” on the contemporary world order [4]. Indeed, its impacts have already cut across the multiple, interconnected and often interdependent systems in the world - notably in the public health, politics, socioeconomic, environmental, and food chains [5]. National healthcare systems of countries were functionally incapable of responding to the pandemic solely by their own capacity, and in just four months into the global outbreak, the WHO had to take a coordinated lead to produce and publish 130 guidance documents on various aspects of preparedness and responses to the pandemic [6].

One of the major impacts of the COVID-19 pandemic over the last year has been an increase in the consumption of antibiotics [7]. Such a sharp increase in the consumption of antibiotics further fuels the challenges posed by antimicrobial resistance (AMR), which has hitherto been characterized by the WHO as the greatest threat to global health [8].

This COVID-19 pandemic should be a powerful awakening call underpinning a compelling reality that global collaborations would be the most, if not the only, effective strategy to tackle such a global threat today. Data should be collected and analyzed in every country and continent as to how healthcare responses to the COVID-19 pandemic affected AMR epidemiology, antimicrobial stewardship (AMS) strategies, programs, and systems. Such global collaborations should stimulate and support scientific research studies and publications across the globe, as is often recommended for such global pandemics [9]. Moreover, such studies about the impacts of COVID-19 on AMR would even be more imperative in Africa where data on antibiotic resistance patterns are scarce, conditions for transmission of deadly infections are optimal, and resources for clinical care are meager.

The overarching impact of the COVID-19 pandemic on AMR in high-income countries has recently been disputed. On one hand, some researchers argued that given the public health interventions made by governments across the globe in response to the pandemic, COVID-19 will not have a significant impact on the evolution of AMR [10]. On the contrary, others argued that regardless of the public health interventions, the impact of the pandemic on AMR is going to be significant [11]. There is a lack of studies about this in low and middle-income countries.

In Africa, the first case of COVID-19 was detected in Egypt on February 14, 2020 [12], about a month before the WHO declared it a global pandemic, while the last case was detected in Lesotho about three months later [13]. As per the latest data from the African Union’s health monitoring portal on COVID-19, Africa CDC, updated by the 9th of April 2021, there are a total of 4.3 million confirmed cases in Africa of which are about 115,000 deaths, 3.9 million recoveries, and the rest being active cases [14]. From these, the number of confirmed cases and deaths per 100,000 population in Africa by the 9th of April 2021 would therefore be 332 and nine, respectively.

The true picture of the epidemiology of COVID-19 in Africa or elsewhere in the world will likely emerge only after the end of the pandemic. But at least at this point, the above data portray the African continent, arguably, as “the least affected,” albeit erroneously. Notwithstanding these data, we argue that the following factors might have contributed to informing them:

Underreporting: Healthcare systems in most African countries are underdeveloped. They lack current technological means for accurate health data collection and reporting, struggle with meager resources, and hardly have favorable workplace environments to capture data in the correct quantity and quality. These challenges have been noticed particularly during this season of COVID-19 [15].

Demographic factors: The median age of Africa’s population is 19.7 years, which makes Africa the youngest continent on the planet. A study showed that the crude fatality rate of COVID-19 patients above 70 years exceeds those below 20 years by about 625 folds [16].

The hygiene hypothesis: This hypothesis in medicine states that early childhood exposure to particular microorganisms could protect against allergic diseases by contributing to the development of the body’s natural immunity [17]. In a way, this might have had also helped Africa.

In this paper (posted earlier on May 7, 2021, at a preprint server: www.preprints.org), we attempt to synthesize early evidence by integrating findings from peer-reviewed articles and also from the gray literature on the likely impacts of the COVID-19 pandemic on AMR epidemiology in Africa over the first year of the pandemic.

## Review

Methodology

A scoping review method [18] was employed to collate and curate resources in the literature. Literature resources were not bounded by geography. Mixed approaches of extracting data (qualitative and quantitative), as per the methodological suggestions by Health Evidence Network [19], were used in synthesizing the evidence. Studies included in synthesizing evidence were identified using PubMed/Medline, ScienceDirect, and Google Scholar. Accordingly, articles dealing with the interactions between COVID-19 and AMR, since the former was declared a global pandemic by the WHO in March 2020, were searched and identified. The key terms used in searching the literature were COVID-19 impacts as combined with antimicrobial resistance, antibiotic resistance, antimicrobial use, and antibiotic use.

Inclusion/exclusion criteria

**Figure 1:**
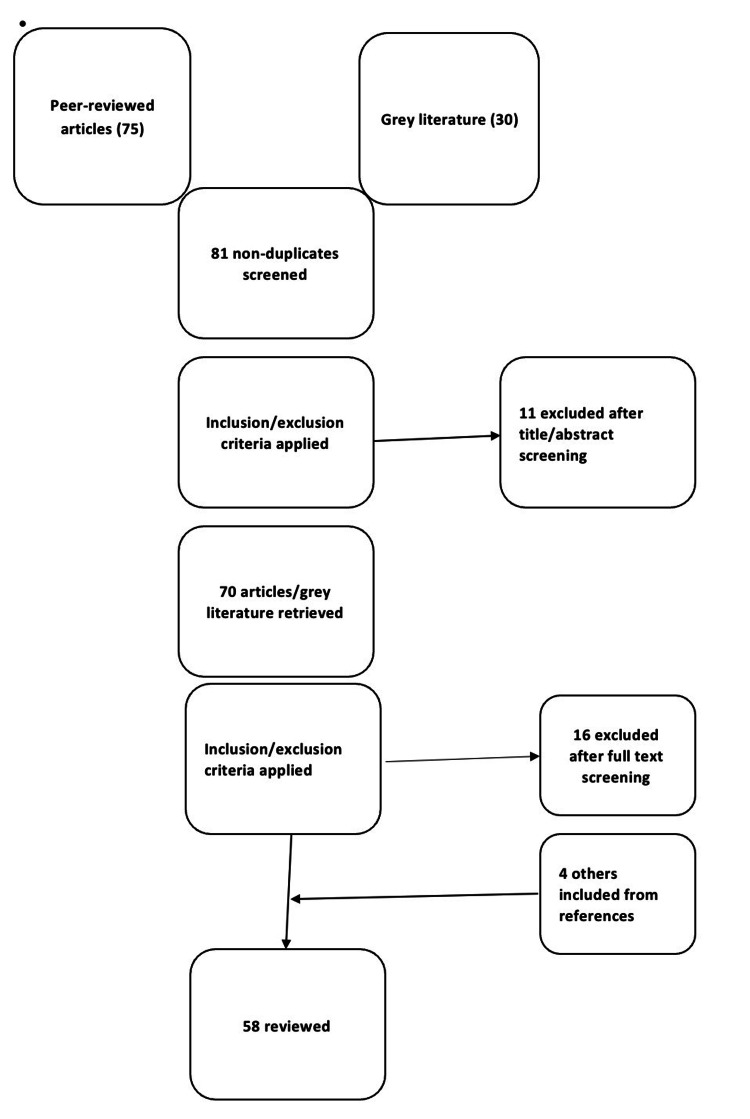
PRISMA-ScR flow diagram of literature selection. PRISMA-ScR, Preferred Reporting Items for Systematic Reviews and Meta-Analyses extension for scoping reviews.

Only articles published in the English language were included. Studies that separately dealt with either COVID-19 or AMR alone were excluded. Instead, only those considering both COVID-19 and AMR with either a stated or implicated aim to look into the clinical or pharmacoepidemiologic interactions between the two were included. Five other studies published before or after the outbreak of the pandemic, including those on the socio-economic impacts of COVID-19, which were found to be relevant in informing the background assumptions and understandings in synthesizing the evidence were also included from references. Figure 1 below shows the Preferred Reporting Items for Systematic Reviews and Meta-Analyses extension for scoping reviews (PRISMA-ScR) flow diagram of literature selection proceedings.

Results and discussion: synthesis and interpretations of the evidence

To put our points in context as to how the COVID-19 pandemic is impacting AMR in Africa, we contend that a holistic approach that takes into account the three determinants driving the evolution of AMR epidemiology, as well as the public health interventions put in place by various countries in Africa, is imperative. These three determinants of the evolution of AMR in populations are (1) emergence of antimicrobial-resistant organisms (AROs), (2) transmission of AROs, and (3) population-level burden of infections caused by AROs [20].

There could be several factors that interact with these three determinants of AMR, either positively or negatively. For the purpose of presenting some contextualized analyses in this paper, such factors have been categorized into two major groups, per the suggestions by Knight et al. [20]: (1) those driven by the outbreak as well as community transmission of COVID-19 and (2) those related to the public health interventions made in response to the COVID-19 pandemic. The interactions that the two categories of factors have with the determinants of AMR evolution are dialectical. Specific factors which can be enlisted under the first category as directly linked to the outbreak and community transmission of the pandemic include increasing antibiotic use, disruption in pharmaceutical supply chains, disruption in access to healthcare services, rising poverty, and inequality within the population. On the other hand, those factors interacting with the three determinants as related to the public health responses put in place by governments to halt the spread of COVID-19 include travel restrictions (domestic/international), physical distancing, closing schools, businesses, and government offices, employment of enhanced infection prevention and control measures in healthcare settings as well as in the community, and setting up centralized rapid response systems to COVID-19 pandemic. The common measures taken to enhance infection prevention and control include frequent hand washing, using biocidal hand sanitizers, environmental disinfectants, and wearing face masks.

To gauge the overall impact of the pandemic on the epidemiology of AMR in Africa, taking a closer look at how specifically these various factors could interact with those determinants - individually or in concert - and examining the overarching outcome of these interactions within the wider context of healthcare and community settings in Africa would be imperative [21].

**Figure 2:**
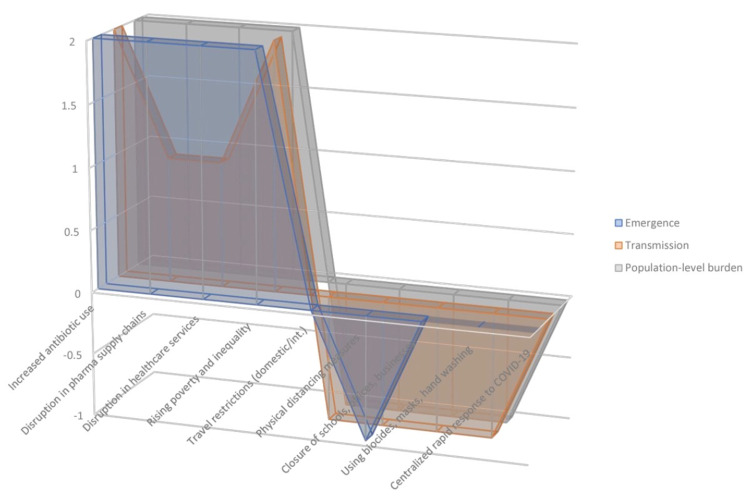
Model constructed based on the opposing interactions that specific factors driven by COVID-19 pandemic (shown here by areas of the geometric figures on the positive side of Y-axis) and those factors driven by the public health responses to COVID-19 pandemic (shown by the area of the geometric figures on the negative side of Y-axis) are having with the three determinants of AMR evolution. Assumptions and designations about the effects of the specific factors on the determinants in constructing the model are: (a) no effect = 0; (b) positive effect (moderate) = 1; (c) positive effect (strong) = 2; (d) negative effect (moderate) = −1; and (e) negative effect (strong) = −2.

Our take on the overarching impacts that COVID-19 is likely having on the epidemiology of AMR in Africa is anchored on a model that we constructed based on the synthesis of early evidence available. Accordingly, computer-based simulations of the areas of the geometric figures constructed for the cumulative impact of the above factors favoring and disfavoring the evolution of AMR in the model show that the former outpaces the latter by no less than 300% (see Figure 2).

Below, therefore, we base our assumptions in this model to examine and discuss such overarching outcomes of these interactions.

Increasing Use of Antibiotics

The rise in the emergence of AMR, secondary to the increasing use of antibiotics after the outbreak of COVID-19, has been of great concern globally [22,23]. Following the outbreak of the COVID-19 pandemic in March 2020, a sharp rise in antibiotic prescriptions has been documented in different parts of the world [7,24-26]. At the level of a specific product, for instance, up to 400% increase in consumption of azithromycin has been reported during this pandemic season [27]. Such sharp rises in the consumption of antibiotics during this pandemic season are being driven, among others, by the prescriptions of antibiotics for empirical treatments, for treatment of health facility-acquired infections (HAIs), and also for the treatment of post-COVID-19 complications, particularly during the recovery stage. We briefly discuss these factors below.

For empirical treatment of COVID-19 and secondary co-infections: Antibiotics were excessively used for empirical treatment in COVID-19 patients, particularly during the early periods after the outbreak of the pandemic [28]. Such empirical uses of antibiotics for the clinical management of COVID-19 have been in the official treatment protocols put in place in response to the pandemic in most countries in line with WHO’s interim guidelines for the clinical management of suspected COVID-19 cases [29]. The macrolide azithromycin has been rapidly repurposed for empiric treatment of COVID-19 due to its extended antiviral effect [30]. Besides, suspected bacterial or fungal co-infections with the coronavirus can also be the major drivers of the rising antibiotic consumption during this season of the COVID-19 pandemic [31,32]. This fits well particularly into resource-limited settings in Africa where basic facilities to perform laboratory screening for specific bacterial co-infections are not only hardly available but also testing services for COVID-19 are lacking in most healthcare facilities. In Ethiopia, for instance, only 72 facilities provide COVID-19 testing services for a population of about 110 million [33]. But such high consumption of antibiotics for secondary co-infection has also been the case in most parts of the globe. It seems this practice was partly influenced by the earliest and most influential studies like the one from China [34] and also of prior pandemics in history [35], which reported that half of the patients who died of COVID-19 and the 1918 “Spanish flu” pandemic had secondary co-infections.

What is more, the potential synergy between viral and bacterial pathogens in exacerbating infections also appears to be at play in driving decisions to frequently use antibiotics in suspected secondary co-infections as that seems to be supported by pathophysiological evidence. Studies have shown that respiratory viral infections may affect the innate immunity of the respiratory system thereby promoting bacterial proliferation, and also increasing the growth of macrophages due to the increased burden of apoptotic cells [36]. Details on this pathogenesis as driven by such effects on innate immune functions likely leading to the synergy between co-infections caused by viral and bacterial pathogens have been discussed elsewhere [37].

For treatment of HAIs: Patients with severe symptoms of COVID-19 infection acquire HAIs, more often than not least, because they stay on mechanical ventilators in healthcare facilities for some time, and it will be very likely that they acquire such infections and hence need antibiotics [38,39]. The possible shortage of mechanical ventilators in most settings in Africa might be cited as the reason to downplay the role of HAIs in boosting antibiotic use. Notwithstanding the paucity of data on the extent of mechanical ventilators available for use in Africa, such an argument cannot water down the ground reality that HAIs are also key drivers of increasing antibiotic consumption in healthcare settings in Africa [40].

For treatment of patients in post-COVID-19 complications: COVID-19 could cause extensive damages to lung tissues and their innate immunity due to a condition driven by the devastating effect of immune dysregulation called cytokine storm [41]. Cytokine storm can be triggered by severe viral infections, like COVID-19, and it may lead to a host of complications thereby leaving recovering patients highly susceptible, including to secondary infections [42]. This, therefore, is also one of the factors driving the increasing use of antibiotics in this season of the pandemic. Here, optimum ground realities for this case, including malnourished and immunocompromised patients, high burden of infectious diseases, insufficient infection prevention and control mechanisms, lack of clean water, etc., aggravate this problem in Africa and therefore further promote the increased use of antibiotics in this pandemic season [43].

What is more, the impacts of increasing antibiotic use on the transmission of AMR could theoretically be arrested by factors related to the public health responses to the pandemic like travel restrictions, physical distancing, closure of schools and businesses, wearing face masks, and frequently washing hands and using biocidal hand sanitizers or environmental disinfectants. But in most settings in Africa, such measures might not have been strictly and properly implemented - due to a number of practical limitations - even when officially declared [12,43]. This reality, therefore, offsets the intended benefits of those public health interventions in Africa. As a result, it can be argued that the COVID-19 pandemic is possibly unhindered in driving the population-level burden of AROs as well in Africa.

Disruption of Pharmaceutical Supply Chains

Most countries in Africa import their essential medicines, including antibiotics, from abroad. For instance, countries like Ethiopia and Sudan import about 80% and 70% of all the pharmaceuticals they need, respectively [44,45]. Given the fact that the outbreak of the COVID-19 pandemic brought the global supply chains of goods and services essential to a standstill, the disruption of the supply chains in the international pharmaceutical trade will likely result in a shortage of antibiotics in most African countries. Such lack of access to antibiotics in situations where the outbreak of the COVID-19 pandemic is further fueling the consumption of antibiotics may bring about a knock-on effect on the demands of antibiotics in healthcare settings and also in the community. In fact, this knock-on effect on the demands of antibiotics coupled with cuts in the production lines at pharmaceutical manufacturers due to the outbreak of the pandemic created a shortage of medications in the market, as reported elsewhere [46]. This in turn likely enhances the circulation of substandard or counterfeited antibiotics, the illegal supply chains of which had hitherto been established more in Africa [47]. Such increasing use of substandard and counterfeited antibiotics in Africa would therefore further fuel the three determinants that drive the epidemiology of AMR: emergence, transmission, and population-level burden of AROs.

In addition, disruption in the supply chains of pharmaceuticals in Africa exacerbates the already dire conditions of AMR by disrupting the continuous supply of regimens to patients hitherto on antimicrobial treatments. Patients whose supply of antimicrobial regimens was disrupted due to the pandemic would either discontinue their treatments or else resort to purchasing counterfeited or substandard products from the black market. Whichever way patients would have to respond to the disruption of their regimens, the end result is one and the same, i.e. treatment failure. Treatment failures due to discontinuation of regimens and/or the use of counterfeited drug products ultimately lead to the emergence of AMR [48]. The prevailing optimum conditions for its transmission in Africa subsequently enhance the burden of drug-resistant infections in the population.

Disruption of Access to Healthcare Services

The other immediate impact of the outbreak of COVID-19 in most parts of the world has been the disruption it caused to the provision of regular clinical and non-clinical services in most healthcare facilities [49]. In most countries, centrally coordinated rapid response systems to COVID-19 were set up following WHO’s declaration of the viral outbreak as a global pandemic. This essentially diverted resources, policy attention, and administrative actions towards such emergency responses to the pandemic, leaving other clinical and non-clinical services disrupted. For instance, WHO’s preliminary analysis on five key essential health service indicators - outpatient consultation, inpatient admission, skilled birth attendance, treatment of confirmed malaria cases, and provision of the combination pentavalent vaccine in 14 African countries - revealed a sharp decline in these services between January and September 2020 compared with the two previous years [50]. The report indicated that during the three months when wider drops in services were observed (May, June, and July), services in the five monitored areas dropped on average by >50% in the 14 countries, as compared to the same period in 2019. In another study in which the damages caused by such disruptions to healthcare services for HIV/AIDS, tuberculosis, and malaria in low-income and middle-income countries were epidemiologically modeled, it was reported that deaths due to the three diseases over the coming five years could increase by up to 10%, 20%, and 36%, respectively, as compared to no COVID-19 pandemic scenario [51]. Moreover, the disruption in healthcare services due to the pandemic also disrupted child vaccination programs against bacterial and viral infections like diphtheria, pertussis, polio, and measles [52]. The pandemic also negatively affected the healthcare-seeking behavior of the population not only in Africa [53] but also in other parts of the world [54]. The updated interim guidelines of the WHO recommend the integration of AMS programs with the clinical management of COVID-19 [55], but even in countries with advanced healthcare systems, antimicrobial stewardship programs are reported to have already become “COVID’s casualty” [56,57]. There is no rationale to think of this impact to be otherwise in the very few African countries where scanty stewardship programs had been reported earlier [58-60]. The cumulative outcome of such impacts of the pandemic would be a rise in the burden of infectious diseases in the general population, which in turn fuels the emergence and spread of AMR in Africa.

Rising Poverty and Inequality

COVID-19 pandemic and the public health policy interventions to halt the spread of the virus have taken a dramatic toll on the global economy [61]. The Sub-Saharan Africa region is said to be one of the hardest hit by the socio-economic impacts of the pandemic [62]. The outbreak of the pandemic dried up the inflow of remittances, essentially freezing the tourism industry and also foreign direct investment in the Sub-Saharan Africa region.

According to an early bird’s eye view study conducted by the United Nations Conference on Trade and Development, the gross domestic products (GDP) of countries in Africa could contract by as high as 7.8% due to the impacts of the COVID-19 pandemic [63]. Moreover, total merchandise exports of the countries could decrease by an average of about 17%. Another study - a survey commissioned by the World Bank - on the socio-economic impacts of COVID-19 in four African countries (Ethiopia, Malawi, Nigeria, and Uganda) reported that an estimated 258 million people - accounting for nearly 80% of the whole populations across the four countries - have lost income due to the pandemic. This study found out that about 20% to 25% of households in each of these four countries were unable to purchase essential medicines and food staples due to the devastating economic impacts of the COVID-19 pandemic [64].

If we further focus our analytical lens on Ethiopia as a case study to illustrate the impacts that COVID-19 is having in the socioeconomic arena in Africa, particularly in exacerbating poverty and inequality, we find compelling data that warrant some brief reviews, as summarized in Table 1 [65,66].

**Table 1:** Early evidence on the socioeconomic impacts of COVID-19 in Ethiopia.

Key socioeconomic parameter	Impact of COVID-19 pandemic	Remarks
Trade	About 30% drop in export of goods and services in 2020	Instability and conflicts in the country are certain to have exacerbated this further
	Expenditure on fuel import drops by 50% (from $2.6 billion in 2019 to $1.3 billion in 2020)	Estimate in best case scenario
	Significant loss of revenue at the Ethiopian Airlines	Partly compensated by globally reactivated cargo traffic especially with supply chains of personal protective equipment and COVID-19 vaccines
Remittances	Up to 15% drop in remittances (estimated at $850 million)	Estimate at the upper limit
Foreign direct investment (FDI)	A sharp and sustained decrease in FDI	This must have further been exacerbated by conflicts and instability in the country
Value of Ethiopian currency (Birr)	Depreciation of the birr by over 17% in nominal terms against US dollar by the end of 2020	Depreciation of birr could even be sharper given global and/or regional conditions as well as the external pressures
Inflation	Consumer price index ≥ 20%	
	Inflation in food price ≥ 30%	By the end of 2020
Fiscal expenditure and revenue	Revenue hit hard due to contraction in the economy, fall in trade taxes (import, export)	
Poverty	Over 2 million people fall into poverty	Conservative estimate
	People who depend on handouts for basic survival including food safety nets could increase at least by 70%	Conservative estimate
Jobs	Over 15% loss of employments or livelihoods (≥4 million people by conservative estimate)	Crisis exacerbated by displacements due to conflicts

Increasing Use of Biocides

The sharp increase in the use of biocides for personal hand sanitization as well as environmental disinfection has been the other important impact of the COVID-19 pandemic [67]. Such a sharply rising use of biocides following the outbreak of the pandemic is reshuffling the microbiota [68]. As has been indicated earlier, this has largely been driven by measures taken as part of the public health responses to the pandemic in most parts of the world. Biocides are employed to kill or stop the spread of pathogenic microorganisms. These are antiseptics or disinfectants which often act via some biological or chemical mechanisms. The most commonly employed active agents in those widely used biocides - particularly in Africa - include ethyl alcohol (denatured), hydrogen peroxide, sodium hydrochloride, triclosan, chlorhexidine, quaternary ammonium compounds, and surfactants, among others [69]. In a recent editorial article published in the Bulletin of the WHO, scientific research studies were called for on the short- and long-term impacts of the rising use of biocides on fueling cross-resistance to antimicrobials during this season of the COVID-19 pandemic [22].

Earlier reports established that there exist links between the increasing use of biocides, much like antibiotics, and selective pressure on the development of AMR [70]. For instance, in a review in which the effects of the sub-lethal concentrations of 13 biocidal agents on antibiotic resistance in gram-negative bacterial species were evaluated, Kampf documented details on antibiotic tolerance, complete resistance as well as mechanisms of resistance development, including horizontal gene transfer and pumping efflux. Accordingly, various strains of bacteria adapted to benzalkonium chloride were found to be resistant to some commonly used antibiotics like ampicillin, cefotaxime, and sulfamethoxazole. Moreover, with the other frequently used biocidal agent, chlorhexidine, resistance by multiple strains of gram-negative bacteria against antibiotics like ceftazidime, sulfamethoxazole, tetracycline, cefotaxime, and imipenem were reported. The study also documented cross-resistance to antibiotics with some biocidal agents including triclosan and sodium hypochlorite, among others [70].

The increasing use of biocidal agents as triggered by the knock-on effect of the public health responses to the COVID-19 pandemic in Africa or elsewhere in the world would inevitably enhance the quantities of biocidal agents disposed into wastewaters, recipient rivers, and the wider ecosystem [71,72]. The disposal of large quantities of such biocidal agents into the natural environment brings about an effect of a sword with double edges - both dangerous - depending on their concentrations within the recipient physical environment.

In the first scenario in which biocidal agents would be detected in the recipient environment in some increasing concentrations but while remaining below the minimum threshold needed to kill or inhibit the growth of bacterial colonies present, they increase selective pressure, thereby enhancing the emergence of AMR. On the flip side, if these disinfectants/sanitizers dumped into the recipient environment are detected in quantities above the minimum lethal concentrations, they would likely kill or inhibit the growth of most bacterial species in the environment. This too might not be required as it brings about negative impacts by destroying the useful or/and harmless bacterial species that contribute to keeping the biological balances - the microbiota - within the wider ecosystem [73].

Overall, the increasing use of not only these biocides but also antimicrobials both in healthcare and community settings will certainly increase and further buildup the quantities of these active agents disposed into the environment [71]. It is also likely that the increasing use of disinfectants and sanitizers would continue during this season of the COVID-19 pandemic and even remains so in the post-pandemic era due to compelling policy changes on infection prevention and control protocols in healthcare settings as well as in the community. If the opportunity can be seized, this current threat of crisis cropping up at the nexus between COVID-19 and AMR can be turned into a good chance to redefine the approach to AMS, including through strengthening diagnostic capacity in Africa [74]. An improved data collection approach that transcends beyond passive surveillance, one which is clinically focused, linking risk factors, microbiology, treatment costs, and outcomes, needs to be designed. Sets of other challenges and opportunities that COVID-19 could offer with regard to AMR are discussed elsewhere [75]. And as it goes, we need more rigorous scientific studies that can shed light on the unfolding crisis at the nexus between COVID-19 and AMR epidemiology in Africa and elsewhere in the world.

## Conclusions

The outbreak of COVID-19 in 2020 has contributed to the rising use of antibiotics and biocides, disruptions in pharmaceutical supply chains and healthcare services, as well as rising poverty and inequality, all of which are important contributors in driving the evolution of AMR. Contrarily, another group of factors related to the public health interventions made by governments in response to the pandemic, including travel restrictions, physical distancing, closure of schools/businesses, extensive use of biocidal sanitizers, or washing hands and face-masking, are supposed to have opposite impacts. A model we constructed based on the strengths of these opposing factors by synthesizing early evidence available predicted that, in cumulative terms, those factors favoring the evolution of AMR likely outpace those disfavoring it by no less than three folds in Africa. It, therefore, seems that COVID-19 could be fueling the evolution of AMR almost unhindered in Africa. Due to the recognition of this crisis, concerted efforts for resource mobilization and global cooperation are needed to tackle it. This crisis, which is cropping up at the nexus between COVID-19 and the already dire conditions of AMR in Africa, can only be tackled through concerted global cooperation. Hence, collaborations but not contradictions or competitions between nationalism and globalism can be the way out.
